# The Epidemic of Hip Fractures: Are We on the Right Track?

**DOI:** 10.1371/journal.pone.0022227

**Published:** 2011-07-25

**Authors:** Klaas A. Hartholt, Christian Oudshoorn, Stephanie M. Zielinski, Paul T. P. W. Burgers, Martien J. M. Panneman, Ed F. van Beeck, Peter Patka, Tischa J. M. van der Cammen

**Affiliations:** 1 Department of Internal Medicine - Section of Geriatric Medicine, Erasmus MC, University Medical Center Rotterdam, Rotterdam, The Netherlands; 2 Department of Surgery-Traumatology, Erasmus MC, University Medical Center Rotterdam, Rotterdam, The Netherlands; 3 Consumer and Safety Institute, Amsterdam, The Netherlands; 4 Department of Public Health, Erasmus MC, University Medical Center Rotterdam, Rotterdam, The Netherlands; University of Otago, New Zealand

## Abstract

**Background:**

Hip fractures are a public health problem, leading to hospitalization, long-term rehabilitation, reduced quality of life, large healthcare expenses, and a high 1-year mortality. Especially older adults are at greater risk of fractures than the general population, due to the combination of an increased fall risk and osteoporosis. The aim of this study was to determine time trends in numbers and incidence rates of hip fracture-related hospitalizations and admission duration in the older Dutch population.

**Methods and Findings:**

Secular trend analysis of all hospitalizations in the older Dutch population (≥65 years) from 1981 throughout 2008, using the National Hospital Discharge Registry. Numbers, age-specific and age-adjusted incidence rates (per 10,000 persons) of hospital admissions and hospital days due to a hip fracture were used as outcome measures in each year of the study. Between 1981 and 2008, the absolute number of hip fractures doubled in the older Dutch population. Incidence rates of hip fracture-related hospital admissions increased with age, and were higher in women than in men. The age-adjusted incidence rate increased from 52.0 to 67.6 per 10,000 older persons. However, since 1994 the incidence rate decreased (percentage annual change −0.5%, 95% CI: −0.7; −0.3), compared with the period 1981–1993 (percentage annual change 2.3%, 95% CI: 2.0; 2.7). The total number of hospital days was reduced by a fifth, due to a reduced admission duration in all age groups. A possible limitation was that data were obtained from a linked administrative database, which did not include information on medication use or co-morbidities.

**Conclusions:**

A trend break in the incidence rates of hip fracture-related hospitalizations was observed in the Netherlands around 1994, possibly as a first result of efforts to prevent falls and fractures. However, the true cause of the observation is unknown.

## Introduction

Fall incidents and fall-related injuries among older people are a major public health problem in ageing societies worldwide [1], [2], [3]. Of people aged ≥65 years approximately one third fall each year [4], [5], [6], [7]. Especially older individuals are at an increased risk of sustaining fractures after a low energetic trauma, *e.g.* a fall incident, due to underlying medical conditions, especially osteoporosis [8].

Osteoporosis, a highly prevalent condition in the older population, is characterized by low bone mass and micro-architectural deterioration of bone tissue. Osteoporosis results in an increased bone fragility and increased susceptibility to fractures [8]. Typical sites of osteoporotic fractures include those of the hip, wrist, vertebrae, and upper arm [9]. Approximately 85% of all hip fractures occur in individuals aged ≥65 years [10]. Hip fractures are, more than any other type of fracture, associated with a loss of independence [11], morbidity [12], and mortality [13].

Besides the health impact on the individual patient, the socioeconomic impact of osteoporosis and of hip fractures in particular is substantial [14]. Hip fractures are currently leading to nearly half (46%) of all injury related healthcare costs in older adults in the Netherlands [15], [16]. In a global perspective, the annual estimated worldwide direct and indirect costs of hip fractures amounted to $34.8 billion in 1990, and are expected to rise to an estimated $131 billion by 2050 [17]. With the expected continuing ageing of populations worldwide [18], it might be expected that the number of hip fractures will increase accordingly, making it necessary to prepare our healthcare systems for this burden.

In order to optimize healthcare use and healthcare planning in an ageing society, accurate numbers in hip fracture incidence are mandatory. The aim of this study was to provide secular trends of age- and gender specific numbers, incidence rates and length of hospital stay (LOS) of hip fractures in the older Dutch population.

## Materials and Methods

For this study all data of hospital admissions due to a hip fracture in persons aged ≥65 years were collected from 1981 throughout 2008 in the Netherlands. The data were retrieved from Statistics Netherlands (CBS, The Hague, The Netherlands), which combines information of the National Medical Registration (LMR) [19] and the National Hospital Discharge Registry. Data regarding hospital admissions, admission diagnosis, LOS in days, age, and gender are stored in this database. The LMR database has a high nationwide coverage and nearly all admissions are stored in this database (less than five percent missing). Hospital admissions data and population numbers were verified with the national Birth-Registry [19]. The Birth-Registry is used to identify individual patients in the National Medical Registry. Data were corrected for missing values by the Statistics Netherlands, and extrapolated to full national coverage [20]. A uniform classification and coding system is used by the LMR for all hospitals and did not change during the study period. Official coding clerks register the diagnosis and injury mechanism of all hospital admissions, based on data obtained from medical records. Throughout the study period, a hip fracture was defined by using the International Classification for Diseases, 9^th^ revision of the World Health Organization, code 820. Older persons were defined as persons aged 65 years and older. Demographic numbers were retrieved from the Statistics Netherlands. In this study the mid-year population was used. The medical ethical review board of the Erasmus MC, University Medical Center, Rotterdam, approved the study (MEC-2010-402) and provided a waiver for ‘informed consent’, because the data were retrieved from a large public accessible database, containing anonymous data on admissions, which cannot be traced to individuals.

Numbers of hospitalizations due to hip fractures were specified for age and gender. The age-specific incidence rates were calculated in 5-year age groups using the number of hip fractures in that specific age group, divided by the population size within that specific age-group for male and female patients, and was expressed per 10,000 persons in that age-group. Age-adjusted incidence rates allowed us to compare the incidence rate for a standardized population during the study period, and were performed by ‘Direct Standardization’ to correct for demographic changes throughout the study period. Growth in the numbers of hospital admissions and LOS were calculated in percentages compared to the index year 1981.

Data were analyzed using a Poisson regression analysis for annual growth in overall hospital admissions for older persons, corrected for population size and age composition. In order to model the trend in hospital admissions, a linear regression model with Poisson error and log link was built with log (mid year population size of each year of the study) as offset factor. To assess if the annual growth changed during the study period for both genders, the Joinpoint Regression Program, Version 3.4.3. (Statistical Research and Applications Branch, National Cancer Institute, USA) was used. This program showed the necessity for assuming a spline instead of a simple linear model, for men and women separately, and determines where to place the knot. The spline function accommodated two piecewise linear fits, connected with one another at the knot. Comparison of these two periods enabled us to detect and quantify changes in the secular trend in admission rates such as stagnation or an increase in admission rates. The best knot was found to be January 1, 1994. The parameter for calendar year, corrected for gender and age-group was transformed into Percentage Annual Change (PAC). The analysis including splines yielded estimates of annual changes in admission rates within each period (1981–1993 and 1994–2008). All statistical analyses were performed using the Statistical Package for the Social Sciences (SPSS) software (version 16.1.1). A p-value <0.05 was considered statistically significant.

## Results

During the study period from 1981 throughout 2008, 355,320 patients aged ≥65 years were admitted due to a hip fracture in the Netherlands. The annual number of hip fracture-related hospitalizations doubled in both men and women, from 7,614 cases in 1981 to 16,049 cases in 2008 (Table 1). The male∶female ratio remained 1∶3 throughout the study period. The crude incidence rate increased, from 46.4 per 10,000 older adults in 1981 to 66.5 per 10,000 in 2008 (an increase of 43.3% compared to 1981), and peaked in 1995 (70.4 per 10,000 older adults). For older men the crude incidence rate increased from 27.6 to 39.5 (an increase of 43.3%) and for older women from 59.5 to 86.8 (an increase of 46.0%) from 1981 to 2008 respectively.

**Table 1:** Population Characteristics of Persons Aged ≥65 Years, Number, Incidence and mean Admission Duration of Hip Fracture-Related Hospitalizations in Persons Aged ≥65 Years (The Netherlands, 1981–2008).

Characteristic	1981	1986	1991	1996	2001	2006	2008
Population ≥65 year (*1,000)	1,642	1,769	1,934	2,061	2,175	2,330	2,415
Female (%)	59.0%	61.2%	60.2%	59.8%	58.9%	57.6%	57.0%
Admissions overall (n)	7,614	9,958	12,565	14,508	14,810	15,249	16,049
- men (n)	1,857	2,281	2,879	3,326	3,385	3,845	4,105
- women (n)	5,757	7,677	9,686	11,182	11,425	11,404	11,944
Incidence rate†	46.4	56.3	65.0	70.4	68.1	65.4	66.5
Mean admission duration, day	37.0	32.1	30.0	23.8	23.1	15.4	14.0

† Crude incidence rate, expressed per 10,000 older adults.

Gender and age-specific incidence rates of hip fracture-related hospital admissions are shown in Table 2. For men and women aged 65–74 years the age-specific incidence rates of hip fractures did not change significantly when comparing 2008 to 1981. However, a strong increase (>50%) in the incidence rate of hospital admissions due to hip fracture was seen in men aged ≥80 years since 1981, up to an increase of 127% in men aged ≥95 years (from 156.3 per 10,000 in 1981 to 354.7 per 10,000 in 2008). Age-specific incidence rates for women aged ≥75 years showed growth of one sixth to a quart.

**Table 2:** Incidence Rates of Hip Fracture-Related Hospital Admissions per 10,000 Persons in the Older Dutch Population (1981–2008).

	65–69 year		70–74 year		75–79 year		80–84 year		85–89 year		90–94 year		≥95 year	
Year	Men	Women	Men	Women	Men	Women	Men	Women	Men	Women	Men	Women	Men	Women
1981	10.2	16.6	16.6	29.5	31.9	58.8	57.7	113.1	89.7	209.8	156.2	272.4	156.3	319.4
1986	11.0	19.6	20.3	34.6	34.8	66.9	65.1	128.6	107.3	229.7	190.0	303.5	233.7	349.2
1991	12.2	21.7	20.7	40.0	41.9	78.9	77.9	134.0	141.1	235.5	220.4	369.9	296.4	379.3
1996	12.2	21.8	24.4	45.5	45.6	82.0	86.3	147.2	148.1	240.0	211.0	363.5	269.0	410.9
2001	9.1	18.0	18.4	38.3	45.1	81.5	80.9	144.6	148.3	241.4	237.3	338.1	345.1	369.3
2006	9.8	15.8	16.9	32.5	38.2	72.1	85.3	138.2	159.6	229.7	265.5	326.1	372.3	385.1
2008	9.1	16.8	18.1	32.2	38.9	70.1	81.1	139.3	161.6	237.5	275.7	325.5	354.7	388.2
Change*	−11%	1%	9%	9%	22%	19%	41%	23%	80%	13%	77%	19%	127%	22%
(95% CI)	(−24; 5)	(−10; 14)	(−5; 26)	(−1; 20)	(8; 37)	(11; 28)	(25; 58)	(16; 31)	(58; 106)	(6; 21)	(47; 112)	(8; 32)	(55; 233)	(0; 48)

*change is 2008 compared to 1981; 95% CI:, 95% Confidence Interval.

**Figure 1:**
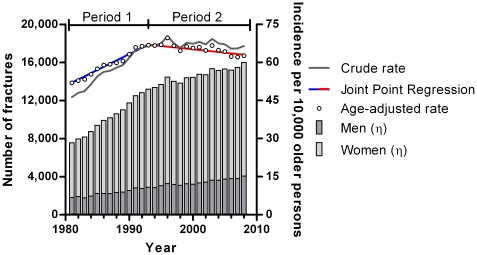
Absolute Numbers, Crude and Age-Specific Incidence Rates of Hip Fracture-Related Hospitalizations in the Dutch Population ≥65 years (1981–2008). Period 1 (blue line): 1981–1993, percentual annual change 2.30%, (95% CI: 2.00; 2.59). Period 2 (red line): 1994–2008, percentual annual change −0.50% (95% CI: −0.70; −0.30).

The overall age-adjusted incidence rate of hip fractures increased (Figure 1) from 52.0 per 10,000 older adults in 1981 to 62.7 in 2008 (an increase of 20.6%). Throughout the study period, the age-adjusted incidence rate for women (68.6 per 10,000 older women in 1981 and 79.9 in 2008) remained twice as high compared to men (27.9 per 10,000 older men in 1981 and 37.8 in 2008).

The PAC, change per year, of the age-adjusted incidence rate was 1.13% (CI 95%: 0.80; 1.45) for men versus 0.52% (CI 95%: 0.24; 0.81) for women over the whole study period. A joint-point regression analysis showed that the change in age-adjusted incidence rates was not constant over time and could be divided into two phases: first, the incidence of hospital admissions due to a hip fracture in older patients increased between 1981 and 1993, and second, decreased between 1994 and 2008 (Figure 1). The annual growth in men was 2.46% (CI 95%: 1.98; 2.94) and in women 2.16% (CI 95%: 1.89; 2.43) in the period 1981–1993. The PAC decreased in the period 1994–2008 to a negative annual growth of −0.34% (CI 95%: −0.86; 0.19) in men and −0.64% (CI 95%: −0.83; −0.46) in women.

**Figure 2:**
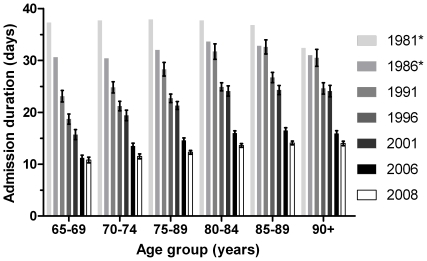
Mean Hospital Admission Duration in Persons Aged ≥65 Years due to a Hip Fracture in the Netherlands between 1981–2008. * No SD data were available before 1991.

**Figure 3:**
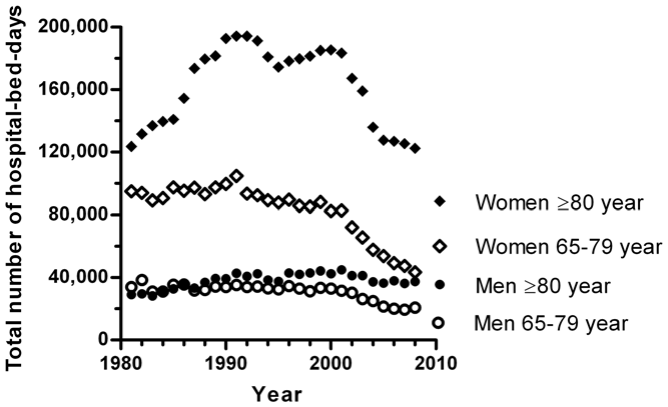
Total Number of Hip Fracture-Related Hospital-Bed-Days in Persons of ≥65 Years in the Netherlands between 1981–2008.

Also the mean LOS decreased throughout the study period in both men and women, from 37.0 days in 1981 to 14.0 days in 2008 (Figure 2). The admission duration decreased over 60% in male and female patients of 65–79 years. Reduction in LOS was smaller in the older patient groups. In patients ≥80 years the LOS per admission was reduced by a third. In general, the LOS was age-related: the higher the age, the longer the admission duration (Figure 2). Although the total number of hip fracture-related hospital admissions increased, the total number of hospital-bed-days decreased due to a reduced LOS per admission. The total numbers of hospital-bed-days are shown in Figure 3 and decreased from 281,396 days in 1981 to 224,002 days in 2008 (a decrease of 20%). For all men aged ≥65 years, the total number of hospital days decreased with 8% (from 62,980 days in 1981 to 58,146 days in 2008). In women aged 65–79 years, a reduction of 54% in hospital days was seen (from 94,903 days in 1981 to 43,474 days in 2008). In women aged ≥80 years the number of hospital days increased until 1991 to 194,264 days and from there on started to decrease, with the total number of hospital days in 2008 (122,382 days) just below (−1%) the total number of hospital days in 1981 (123,513 days).

## Discussion

In order to determine trends in hip fractures in the older Dutch population, all hip fracture-related hospitalizations were analyzed from 1981 throughout 2008. The age-adjusted incidence rates of hip fractures increased until the end of 1993 in the population ≥65 years. After that year, a trend break was observed and the incidence rates started to decrease. Although an encouraging decrease in the age-adjusted incidence rates was observed, the absolute number of hip fractures continued to increase due to a rising number of older persons in the population.

Comparable trends of decreasing incidence rates for hip fracture-related hospitalizations since the mid-nineties have been reported in several countries around the globe, such as the United States [21], Canada [22], and Finland [23]. However, not all findings across western countries are consistent. A recent study from Germany failed to demonstrate a decline in hip fracture incidence rates [24]. Since most studies on hip fracture incidence from multiple countries point in the same direction, there might be a causal explanation for this observation. However, there is no simple answer to this question, because risk factors for hip fractures are multifactorial, as mentioned by Leslie *et al*. [22] Important developments over the last two decades include: the increasing awareness of falls [25], [26], the implementation of guidelines for the diagnosis and treatment of osteoporosis [27], [28], increasing availability and use of bisphosphonates [29], and an improvement of calcium intake and vitamin D status, although the latter is argued by some [22]. Other nationwide changes, such as the prevention and improved treatment of cardiovascular diseases in the general population may also have contributed to the observed trend break. A large Finnish twin-study recently demonstrated that cardiovascular diseases are associated with the development of hip fractures [30]. However, the exact mechanism behind this association is not clear yet [30]. Another possibility might be that general health [31] and bone quality [32] have improved since smoking has been discouraged. The proportion of smokers is decreasing rapidly in the Netherlands [31], [33] as well as in other countries [34], [35]. Furthermore, the Statistics Netherlands (CBS) reported that the mean body weight has increased in the Dutch population [33]. An increased Body Mass Index is associated with a lower fracture risk [36].

A remarkable difference was observed between the younger and older age-groups. Whereas incidence rates decreased in persons <80 years, the incidence rate stabilized in females aged ≥80 years, and continued to increase in males ≥80 years. This finding is worrisome because the population of 80 years and over is the fastest growing segment in the ageing population [37] and because mortality and morbidity associated with hip fractures are greater for the oldest old, and are higher in men than in women in the first year after sustaining a hip fracture [38], [39]. A possible explanation for this observation might be that life expectancy increased more rapidly in men compared to women over the past decades, resulting in a smaller gap in life expectancy between men and women [40]. Consequently, men have become more vulnerable for age-related (co)morbidities, such as osteoporosis and hip fractures, which were previously frequently seen in older women. This assumption is supported by a previous report on a more rapid increase in fall-related injures, hospitalizations, and mortality in older men than in older women in the Netherlands over the past decades [1], [41]. Another possible explanation might be that osteoporosis in men is frequently underdiagnosed and undertreated [42].

The number of hospital-bed-days per admission is considered to be one of the most important determinants of total costs per hip fracture in an individual patient [43]. Therefore, a reduced LOS is necessary in order to reduce hospital care demands and to limit related healthcare costs. During the study period the LOS decreased by two-thirds. Several factors might have contributed to this impressive reduction: the rapid improvement of surgical and anesthetical care over the last decades, resulting in less invasive surgical procedures; the introduction of new hip prostheses, and implants; protocols for early timed surgery after a hip fracture; better pain management and better post-operative care with early mobilization; early discharge to designated rehabilitation places and skilled nursing homes; and the implementation of hip fracture treatment guidelines [44], [45], [46]. In addition, during the final years of the study period a change in the financing structure of Dutch hospitals, which was introduced in 2004, may have led to a further decline in LOS.

A strength of the present study is the availability of population-based in-hospital data, covering a period of 28 years. The Dutch healthcare system is characterized by full health insurance coverage and full accessibility for the whole population during the study period. Since 1981 absolute numbers of hip fracture related hospital admissions and hospital-bed-days in all hospitals in the Netherlands have been recorded with nearly complete national coverage in a highly accurate electronic database. Throughout the study period, the coding system of the National Medical Registry did not change and no major policy changes were introduced in the Netherlands which might have affected the increase in admission rates. However, this study has some limitations. A possible limitation is that these data describe the situation in one country, which may not directly translate to other western countries, because of differences in healthcare system characteristics and demographics. Nevertheless, since hip fracture trends [21], [22], [47] in other western populations are comparable with the trends in the Netherlands, there is no reason to assume that hip fractures trends will be substantially different in other countries. This study is based on a linked administrative database, which does not contain clinical data regarding underlying diagnosis, co-morbidity, injury severity, lifestyle, or medication use of the patients. This limits the interpretation of the causal mechanisms behind the observed trends. Furthermore, readmissions in one calendar year were not excluded and could potentially lead to some ‘double registration’. However, it is unlikely that readmissions influenced our results, since readmissions for injuries constitute at the most 2.6% (at the maximum) in the Netherlands, as was found in a study by Polinder *et al*. [48]

In summary, the increase in hip fracture incidence rates slowed down between 1981 and 1993, and the incidence rates started to decrease over the last 14 years. However, incidence rates nowadays remain higher than in 1981, suggesting that there is still room for improvement. Furthermore, the continuing increasing incidence rates in the oldest men is a worrying trend that deserves specific attention, since the group of persons aged 80 years and older are the fastest growing segment of aging societies. With the expected ageing of societies worldwide, continued attention is needed in order to cope with the demand of hip fracture related care in the near future.
